# “I put it in my head that the supplement would help me”: Open-placebo improves exercise performance in female cyclists

**DOI:** 10.1371/journal.pone.0222982

**Published:** 2019-09-24

**Authors:** Bryan Saunders, Tiemi Saito, Rafael Klosterhoff, Luana Farias de Oliveira, Gabriel Barreto, Pedro Perim, Ana Jéssica Pinto, Fernanda Lima, Ana Lucia de Sá Pinto, Bruno Gualano

**Affiliations:** 1 Applied Physiology and Nutrition Research Group, School of Physical Education and Sport, Rheumatology Division, Faculdade de Medicina FMUSP, University of São Paulo, São Paulo, Brazil; 2 Institute of Orthopaedics and Traumatology, Faculty of Medicine FMUSP, University of São Paulo, São Paulo, Brazil; 3 Department of Biochemistry and Molecular Biology, Federal University of Paraná, Curitiba, Paraná, Brazil; 4 Laboratory of Assessment and Conditioning in Rheumatology (LACRE), Rheumatology Division, Universidade de São Paulo, São Paulo, Brazil; Universidade Federal de Juiz de Fora, BRAZIL

## Abstract

This study investigated the effect of open-placebo on cycling time-trial (TT) performance. Twenty-eight trained female cyclists completed a 1-km cycling TT following a control session or an open-placebo intervention. The intervention consisted of an individual presentation, provided by a medic, in which the concept of open-placebo was explained to the participant, before she ingested two red and white capsules containing flour; 15 min later, they performed the TT. In the control session, the participant sat quietly for 20 min. Heart rate and ratings of perceived exertion (RPE) were monitored throughout exercise, while blood lactate was determined pre- and post-exercise. Post-exercise questionnaires were employed to gain insight into the perceived influence of the supplement on performance. Open-placebo improved time-to-completion (P = 0.039, 103.6±5.0 vs. 104.4±5.1 s, -0.7±1.8 s, -0.7±1.7%) and mean power output (P = 0.01, 244.8±34.7 vs. 239.7±33.2, +5.1±9.5 W) during the TT. Individual data analysis showed that 11 individuals improved, 13 remained unchanged and 4 worsened their performance with open-placebo. Heart rate, RPE and blood lactate were not different between sessions (all P>0.05). Positive expectation did not appear necessary to induce performance improvements, suggesting unconscious processes occurred, although a lack of an improvement appeared to be associated with a lack of belief. Open-placebo improved 1-km cycling TT performance in trained female cyclists. Although the intervention was successful for some individuals, individual variation was high, and some athletes did not respond or even performed worse. Thus, open-placebo interventions should be carefully considered by coaches and practitioners, while further studies are warranted.

## Introduction

A beneficial effect arising from the belief that one has received a positive intervention is known as the placebo effect [1]. The placebo effect is a powerful tool that can be used to modify subjective feelings, such as fatigue and disease symptoms, as well as objective measures of tolerance to pain [2, 3]. These responses can be variable, and are determined by environmental, psychological and neurobiological factors including verbal cues, expectancy, desire, positivity, beliefs and conditioning [4, 5]. Notwithstanding the potential of the placebo effect, ethical considerations are inherent since classical placebo treatment requires a level of deception of the individual and/or the administrator, with potential implications on trust and harm to patients [6]. Interestingly, recent evidence with open-placebo administration suggests that deception may not be necessary to elicit a placebo effect.

Clinical studies investigating the effect of open-placebo (also referred to as non-deceptive or open-label placebo), where participants are deliberately informed that they would receive placebo pills, have shown significant improvements in symptoms for patients with irritable bowel syndrome [7], chronic low back pain, [8], allergic rhinitis [9, 10], depression [11] and cancer-related fatigue [12]. Although the exact mechanisms of how open-placebo might cause these effects are not fully elucidated, positive expectations of the individual appear unnecessary [10]. To date, no study has investigated the effect of open-placebo on exercise performance.

The placebo effect is apparent in sports nutrition, characterised by an improvement in exercise capacity and performance despite having received an inert substance [13–17]. A classic study showed a linear association between improvements in cycling performance and deceptive administration of placebo insinuated to be moderate and high doses of caffeine [15], demonstrating the power of participant belief. Indeed, it is generally considered that individuals must believe that they have ingested the active substance to gain an advantage despite having ingested placebo; this is highlighted by the opposite being true, namely the nocebo effect, where an individual performs worse because they believe (correctly) that they have ingested the inert substance [13] or because they have been informed that the substance is harmful to performance [14, 18] when they have ingested a placebo. However, there is some clear variability in the placebo response since recent evidence has shown that even when a placebo is deceptively administered, a positive performance outcome can occur even when individuals believe they have received a placebo [19]. This suggests that individuals may not need to believe they have ingested an active substance to gain a performance benefit, making open-placebo an interesting area for investigation and potential application in sports.

The aim of this study was to investigate the effect of open-placebo on 1-km cycling time-trial performance. A secondary aim of this investigation was to explore any potential factors relating to individual beliefs and optimism (via questionnaires) that may underpin potential variation in responses. We hypothesised that open-placebo would result in an improved overall cycling time-trial performance, but that there would be some variability in response that could be explained by factors including expectations, positivity and belief in supplements.

## Materials and methods

### Participants

A call for female cyclists willing to participate in a research study was made on social media and within several cycling groups (Facebook and WhatsApp). A total of forty-four females registered their interest and, following assessment of eligibility and availability, thirty were recruited to take part in this randomised, cross-over study. Inclusion criteria included, i) aged between 18 and 45 years old; ii) a minimum of one-year training experience in cycling. Two participants withdrew during data collection (one due to a change in availability and the other failed to respond to further contact) meaning a total of twenty-eight females (Table 1) completed all exercise sessions. Participants were informed of all protocols and risks associated with the study and provided written informed consent prior to participating in the study. The study was first approved by the Ethical Advisory Committee of the School of Physical Education and Sport of the University of São Paulo, and all experiments were performed in accordance with all institutional guidelines and regulations.

**Table 1:** Participant characteristics.

Characteristic		Mean (SD)
Age (y)		36 (6)
Height (m)		1.65 (0.06)
Body mass (kg)		62.5 (7.7)
Experience (y)		3 (2)
VO_2max_	Absolute (L·min^-1^)	3.3 (0.5)
	Relative (ml·kg·min^-1^)	53.2 (7.2)
Weekly training	Duration (h)	16 (6)
	Distance (km)	248 (66)
Number of supplements currently consumed		6 (4)

### Experimental design

Participants attended the laboratory on three separate occasions. During the first visit, participants read and signed the informed consent form before completing several questionnaires (detailed below). They then performed an incremental cycling test for the determination of maximal cycling power output and VO_2max_. Following a 15 min rest [20], participants performed a familiarisation of the main exercise protocol, a 1-km cycling time-trial. The next two visits were for the completion of the main trials, separated by one week.

The main trials were performed in a counterbalanced, randomised and crossover manner and performed at the same time of day for all individuals (between 05:00 and 12:00) to account for circadian variation in performance [21]. Participants abstained from strenuous activity and alcohol and recorded their food intake in the 24 h period prior to the initial main trial and adopted the same dietary intake prior to the next session. Caffeine intake was prohibited on the day of the main sessions. Since all participants were involved in structured training programs, they were requested to maintain identical training routines for the weeks in which they completed the main trials. Adherence to these restrictions was confirmed verbally with the volunteers prior to their second main session.

### Experimental procedures

#### VO_2max_ test

The test was performed on a cycle ergometer (Lode Excalibur, Lode, Lode B.V., Groningen, Netherlands) and began with a load of 50 W, increasing every 3 min by 25 W until volitional exhaustion [22]. Verbal encouragement was provided to aid participants in reaching their maximum. Ventilatory and gas exchange measurements were recorded using a breath-by-breath system (K5, Cosmed, Italy); the highest value averaged over a 15-s period during the test was defined as VO_2max_. Maximal power output was calculated as the last completed stage plus the fraction of time spent in the final non-completed stage multiplied by 25 W. Data was used to characterise the sample population.

#### Open-placebo intervention

All participants were given an individual ~5-min talk in which the concept of open-placebo was explained in detail using a PowerPoint presentation. The presentation was entitled “Possible benefits of open-placebo in sport” and included the following information: 1) Definition of a placebo and the placebo effect; 2) examples of the placebo effect in medicine and sports; 3) explanation of open-placebo; 4) examples of open-placebo in medicine. The presentation ended with three considerations for the athlete prior to taking the capsule: i) believing is important for the placebo effect; ii) however, belief in the placebo effect is not necessary as the effect may be automatic/unconscious; iii) taking the pills is important to obtain an effect. The presentation was delivered in a standardised manner by two trained female medics who wore lab coats throughout [23]. Athletes could ask any questions following the short presentation. Immediately following the individual presentation and 15 min prior to the 1-km cycling time-trial, participants ingested two red-and-white placebo capsules containing 100 mg of flour each (Fármacia Analítica, Rio de Janeiro, Brazil). Thereafter, they sat quietly with the medic and engaged in light conversation. In the control session, no specific information was provided prior to the individuals performing the time-trial, although they remained in the company of the medic for 20 minutes to standardise each session. Athletes were strictly requested not to discuss any study details with each other between sessions to minimise any influence from teammates.

#### 1-km cycling time-trial

All main trials were performed on a road cycling bicycle (Caloi Strada, size medium, Caloi, São Paulo, Brazil) attached to a roller, which was connected to appropriate software (CompuTrainer, RacerMate Inc, South Dakota, USA). The bicycle was calibrated according to manufacturer recommendations before participants performed a 10 min warm-up at 100 W, followed by a 2 min rest period during which they remained quietly seated on the bike. They then performed the 1-km cycling time-trial. Participants were instructed to complete the 1-km protocol as fast as they possibly could. The gear on the bike was fixed (50 x 17) and the participants were not allowed to change it throughout the time-trial to replicate track-cycling. Verbal encouragement was provided by two experimenters who were blinded as to which intervention the volunteers had undergone. Participants were blinded to all performance variables during the time-trial except distance covered and did not receive any performance information until all participants completed the study.

Time to complete the time-trial and mean power output were recorded as exercise performance measures. Heart rate was monitored consistently throughout exercise at a frequency of 5 Hz using a heart rate monitor (H7, Cosmed, Italy) with telemetry data transmission connected to a phone app (Polar Beat). Ratings of perceived exertion were recorded following 50% and 100% of the time-trial using the 6–20 point Borg scale [24]. Finger-prick blood samples were taken immediately pre- and post-exercise for the determination of lactate concentration.

#### Questionnaires

On the first visit to the laboratory, all participants provided information regarding their training and supplementation habits, and completed validated questionnaires on supplementation beliefs [25] and life orientation, specifically validated for Portuguese speakers (TOV-R) [26]. The Sports Supplements Beliefs Scale evaluates an individual’s confidence in supplements by having them respond to 6 statements on a 6-point Likert-type scale ranging from strongly disagree (1) to strongly agree (6); the test can distinguish scores between users (scores >20) and non-users (scores <20) of supplements [25]; a maximum achievable score is 36. The Life Orientation Test was developed to assess optimism [27] and requires individuals to score statements on a 5-point Likert scale from completely disagree (0) to completely agree (4). The higher the score, the greater the degree of optimism; a maximum achievable score is 20. Immediately following the open-placebo session, the athletes were asked to respond in writing to two questions regarding the intervention and their performance. Specifically, question one first reinforced that the supplement they received contained an inert substance before asking them to respond yes or no to the following: “Did you believe that the supplement you received contained an inert substance?” If they answered no, they were asked, and provided space to explain, why. The second question asked them to rate how much they believed the supplement influenced their performance on a Likert scale (0, Nothing; 1, A little; 2, Moderately; 3 A lot; 4 Extremely) and to describe why. The information provided from these responses was analysed and used to explore as potential factors that may underpin potential variation in responses.

#### Blood measurements

Blood lactate was determined from blood plasma samples. A small aliquot (20 μL) of blood was collected from the fingertip into a microtube containing the same volume of an ice-cold 2% NaF solution and homogenized. The samples were centrifuged at 2000 g for 4 min at 4°C to separate plasma from erythrocytes. Plasma was subsequently removed and stored at -80°C until analysis. Plasma lactate was determined spectrophotometrically using an enzymatic-colorimetric method as supplied by a commercially available kit (Katal, Interteck, São Paulo, Brazil).

### Data analysis

All assessed variables were analysed using a Shapiro-Wilk test and visual methods for normality, whilst a Mauchly test was used for homogeneity and variance/sphericity. Paired sample t-tests were used to determine the effect of open-placebo on exercise variables (time-to-completion, mean power output). Blood lactate and ratings of perceived exertion were analysed using a mixed model with individuals assumed as a random factor and intervention (2 levels) and time (2 levels) assumed as fixed factors. Mean and maximum heart rate were analysed using a paired samples t-test.

Individual responses were calculated according to time-to-completion using the spreadsheet of Swinton et al. [28] using 50% confidence intervals, a typical error (0.4202 s) calculated from 1-km time-trial reproducibility data from Bellinger and Minahan [29] and a smallest worthwhile change of 0.2 x the standard deviation of the control session [30]. A Spearman’s Rank-Order correlation was performed to determine the association between the change in time-to-completion during the 1-km with open-placebo compared to control (ΔTTC) and questionnaires scores. After separation into groups according to who improved, did not improve and worsened performance, a Kruskal-Wallis test was performed to determine difference in the response to the questionnaires. All data are presented as mean ± 1SD, unless stated otherwise, and significance level was set at p ≤ 0.05. The analyses were performed using SAS (version 9.3) and SPSS (version 23.0).

## Results

### 1-km cycling time-trial

**Fig 1:**
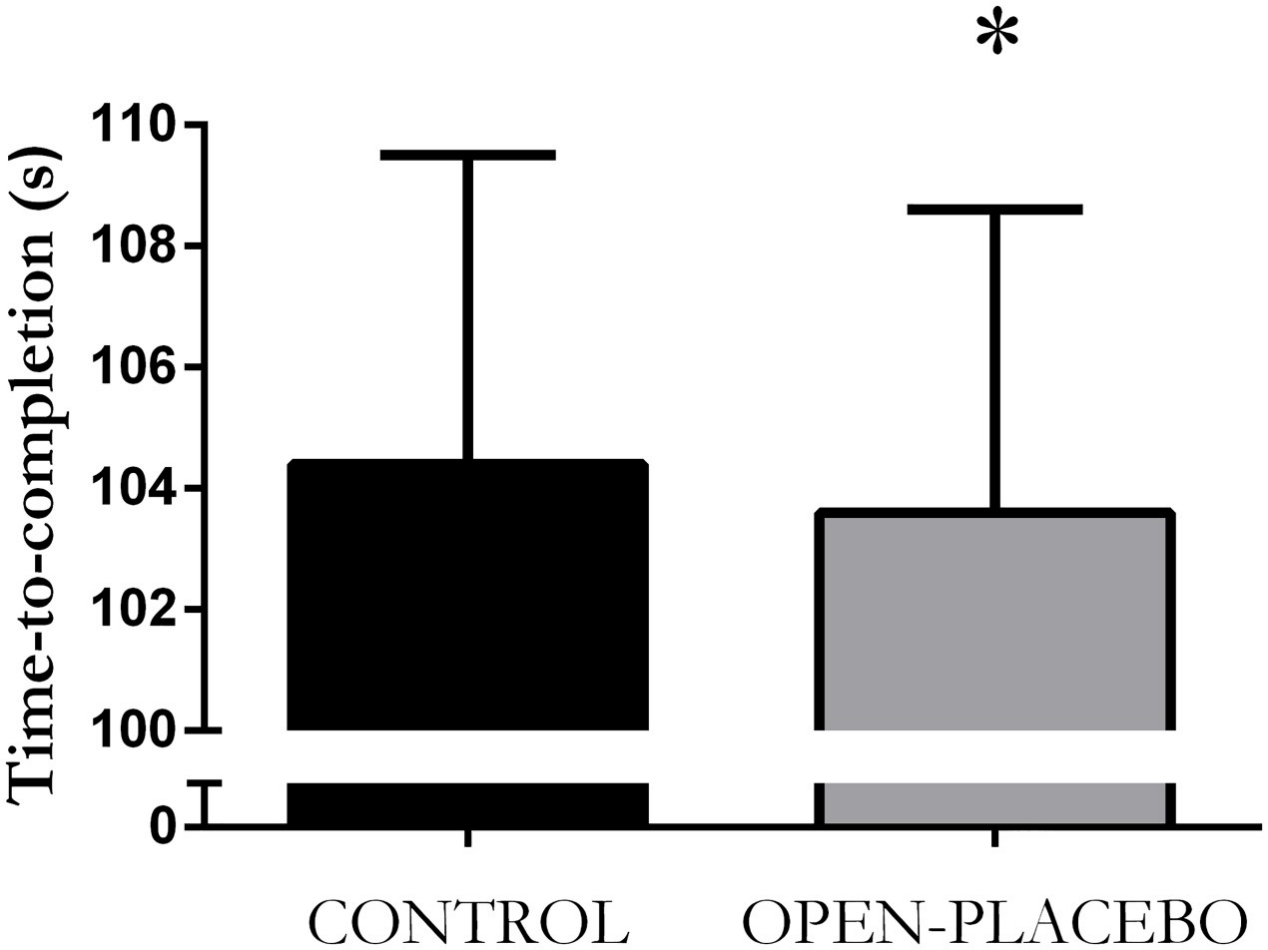
Time-to-completion for the 1-km cycling time-trial during the control (black bar) and open-placebo (grey bar) session. *P = 0.039 from Control.

There was no effect of session order on time-to-completion or mean power output (both P > 0.05). Open-placebo improved time-to-completion (P = 0.039, 103.6 ± 5.0 vs. 104.4 ± 5.1 s, ΔTTC: -0.7 ± 1.8 s, -0.7 ± 1.7%, 95% CI -0.1, -1.4 s; Fig 1) and mean power output (P = 0.01, 244.8 ± 34.7 vs. 239.7 ± 33.2, ΔMPO: +5.1 ± 9.5 W, +1.9 ± 3.9%, 95% CI 1.6, 8.6).

**Fig 2:**
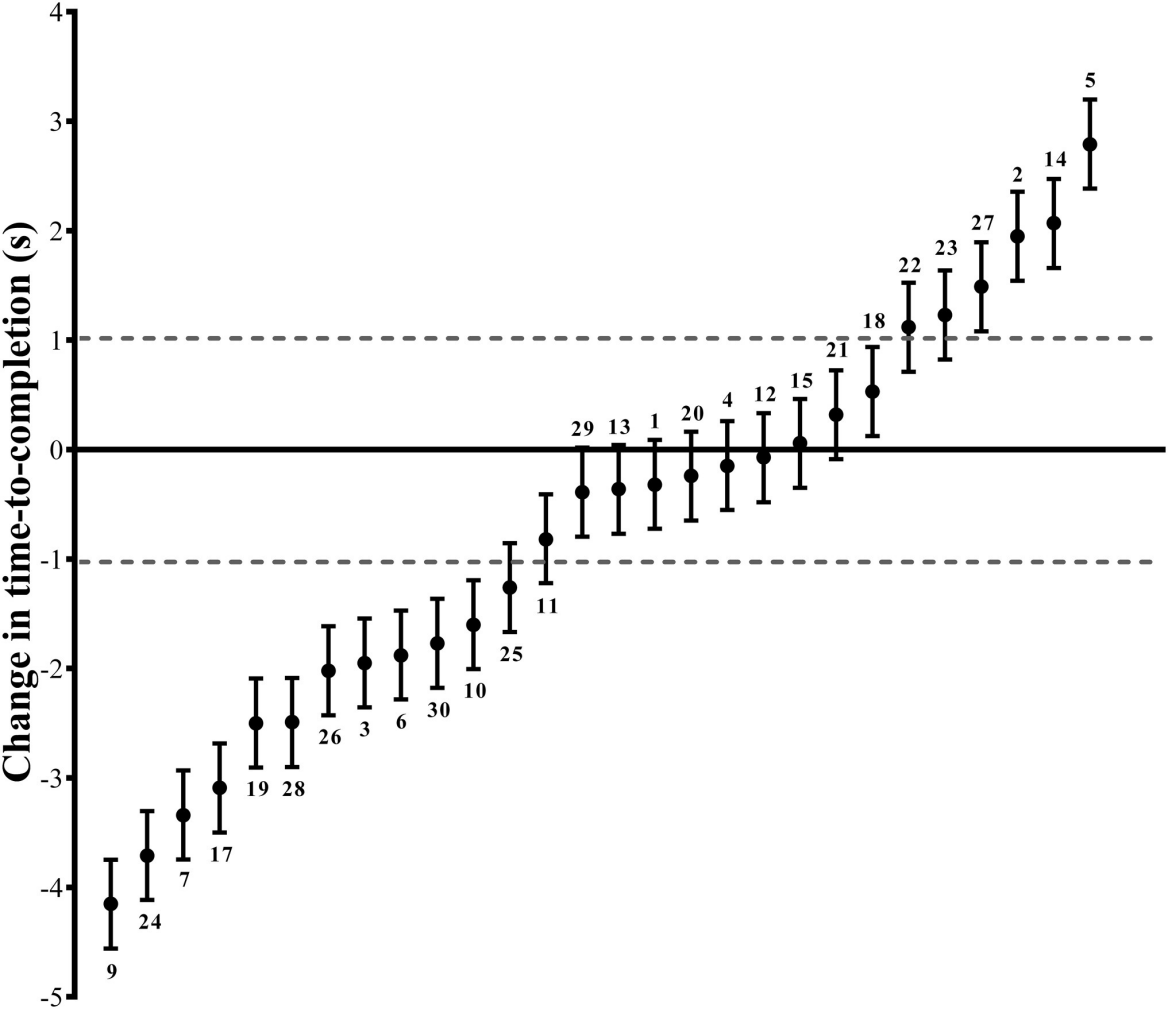
Change in time-to-completion (ΔTTC) of the 1-km cycling time-trial with open-placebo compared to control. Data are individual ΔTTC ± 50% confidence intervals. The dotted grey lines indicate the smallest worthwhile change in performance. Participant numbers are displayed above or below the data points.

Individual data analysis according to the methods of Swinton et al. [28] that determined the number of individuals whose performance changed above the smallest worthwhile difference (±1.02 s) showed that 11 individuals improved performance following open-placebo administration (Fig 2). Four individuals showed measurable decrements in performance and the remaining 13 cyclists showed true score changes and confidence intervals within the limits of the test. Further demonstrating there was no influence of session order on performance, five of those who improved performed the open-placebo intervention first, while the other six performed the control session first.

### Heart rate, blood lactate and perceived exertion

**Fig 3:**
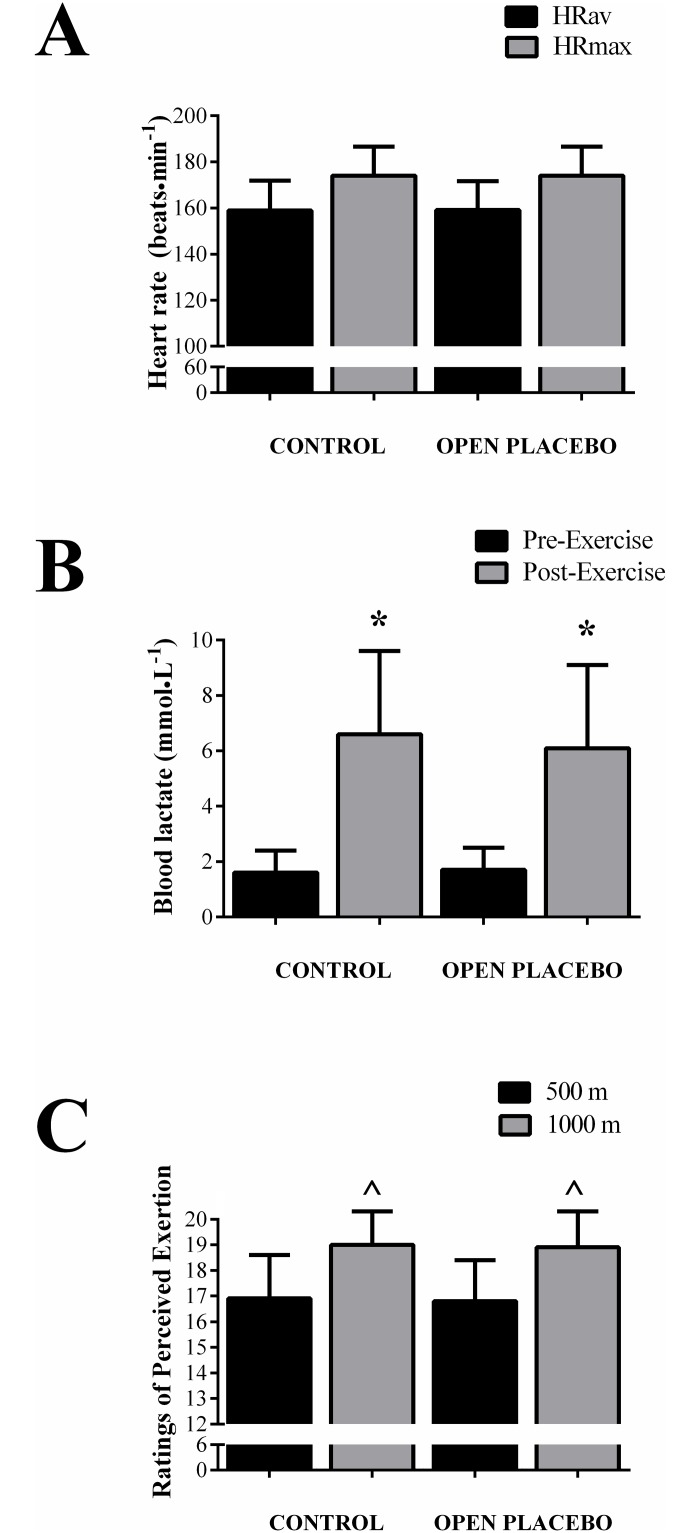
Physiological measures. Panel A: Mean (HRav) and maximum (HRmax) heart rate during the control and open-placebo sessions. Panel B: Blood lactate concentration pre- and post-exercise during the control and open-placebo sessions. *P<0.001 from pre-exercise. Panel C: Ratings of perceived exertion throughout the 1-km time-trial during control and open-placebo sessions. ^^^P<0.001 from 500 m.

Mean (159 ± 13 vs. 159 ± 13 beats·min^-1^, P = 0.81) and maximal (174 ± 13 vs. 174 ± 13 beats·min^-1^, P = 1.0) HR were not different between the open-placebo and control sessions (Fig 3A). There was an effect of time for blood lactate (P < 0.0001), with increased post-exercise lactate concentration compared to pre-exercise, with no differences between trials at any timepoint (intervention x time interaction, P = 0.40; Fig 3B). There was an effect of time for ratings of perceived exertion (P < 0.0001), with higher values at the end of the test compared to the middle, with no differences between trials at any timepoint (intervention x time interaction, P = 0.91; Fig 3C).

### Questionnaires

Two of the 28 volunteers did not believe that the substance they ingested was an inert substance. One of these 2 was the individual who most improved on open-placebo (#9, -4.15 s) and stated she did not believe the substance was inert, “Because of the study, I think it would invalidate the test if it did not contain a substance”. The other individual (#11) who did not believe the supplement contained an inert substance did not provide a reason why; her ΔTTC was -0.82 s. The remaining twenty-six participants responded that they did believe the supplement contained an inert substance.

**Fig 4:**
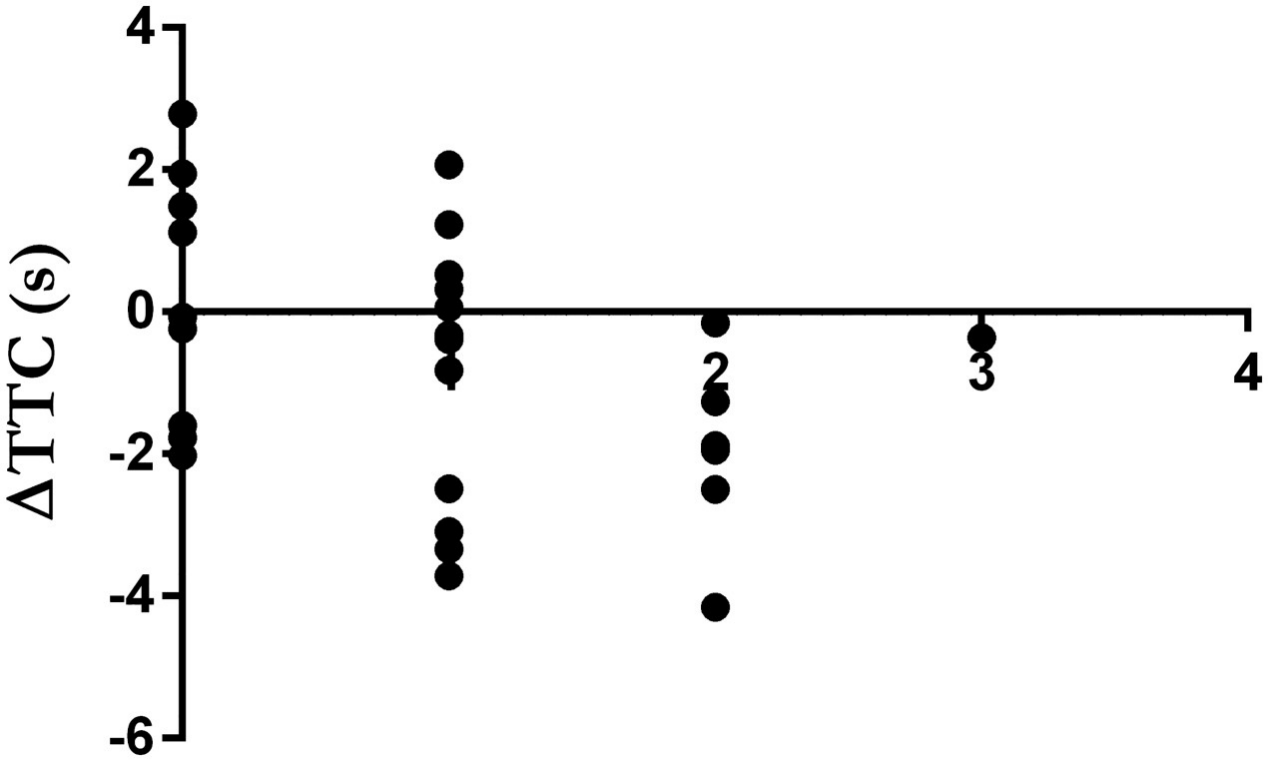
Change in exercise performance from control (ΔTTC; y-axis) in relation to individual belief of how much the supplement influenced exercise performance (x-axis).

**Fig 5:**
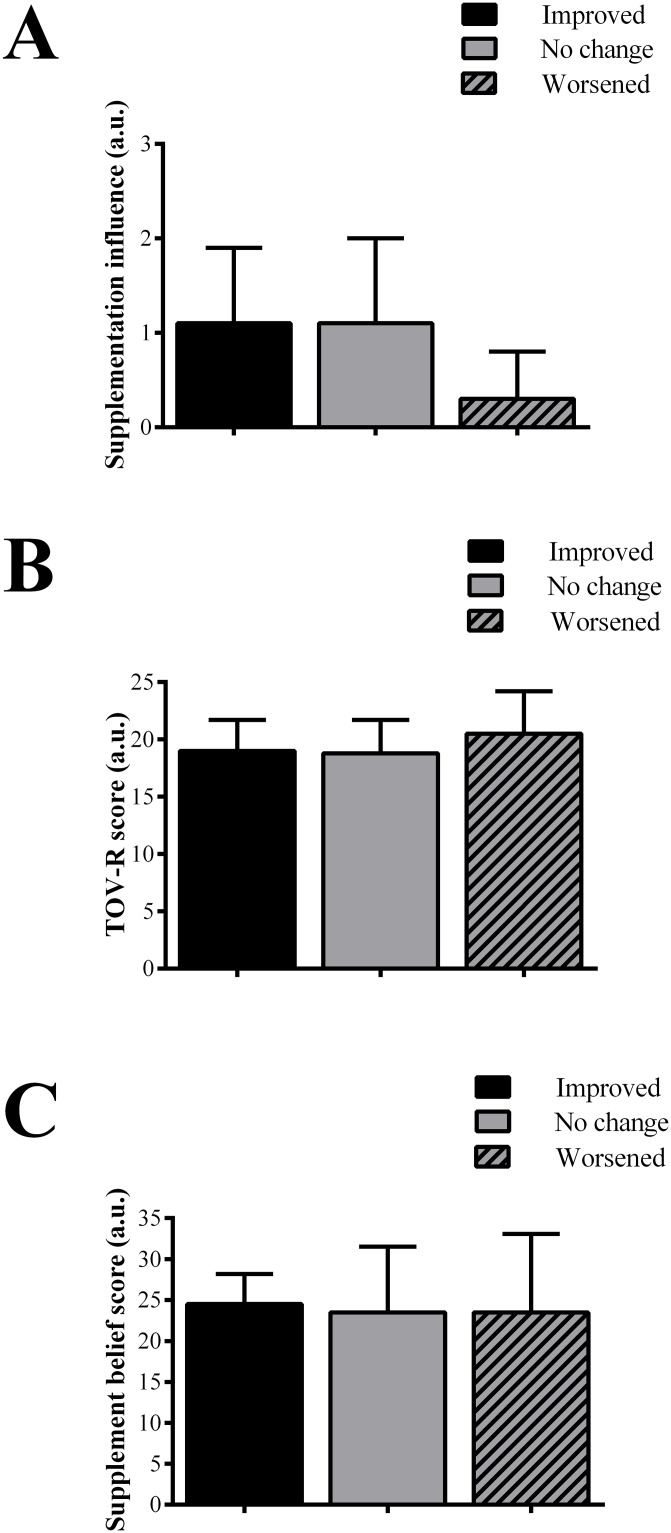
Questionnaire scores according to change in performance. Panel A: Scores for how much the individuals believed the supplement influenced their performance in the open-placebo session for those who improved, did not change, or worsened their performance. Panel B: Life orientation test (TOV-R) scores separated according to those who improved, did not change, or worsened their performance. Panel C: Sports supplement belief scores separated according to those who improved, did not change, or worsened their performance. [A.U. = Arbitrary units].

Nine individuals responded that they did not believe the supplement they had ingested influenced their performance at all {0, Nothing}, 12 reported it influenced their performance a little {1}, 6 reported it had a moderate influence {2} and 1 individual reported it influenced a lot {3}. Life orientation test (TOV-R: 19 ± 3) and sports supplement belief (24 ± 7) scores were high. Change in exercise performance (*i*.*e*., ΔTTC) was significantly correlated with the individual belief of how much the supplement influenced exercise performance (r = -0.393, P = 0.039; Fig 4), but not with TOV-R (r = 0.116, P = 0.56) or sports supplement beliefs (r = -0.052, P = 0.79). When individuals were separated into those who improved, did not change or worsened performance, there were no differences in the responses between groups for how much the supplement influenced exercise performance (P = 0.16; Fig 5A), TOV-R (P = 0.67; Fig 5B) or sports supplement beliefs (P = 0.99; Fig 5C).

Individual responses to how much they believed the supplement influenced performance are reported in Table 2. Several individuals who improved performance reported positive feelings and expectations relating to the open-placebo and exercise (#9, #19, #6, #3), with reports of clear belief in the information provided by the medic (#3) and mental focus on the potential beneficial effects of the placebo capsules (#19 and #6). Nonetheless, some of those who improved reported that either they felt more tired (#24, #28) or that they didn’t notice a difference (#26) and maybe performed worse (#10 and #30), while others even reported that they felt the supplement had very little effect because they knew they had received a pill containing an inert substance (#7 and #17). Altogether, these observations suggest that expectation may not be necessary to improve performance with open-placebo, and that effect is unconscious, at least for some individuals.

**Table 2:** Participant ID, change in time-to-completion of the 1-km cycling time-trial with open-placebo compared to control (ΔTTC), responses to the post-exercise question (Q2) which asked, “How much do you believe the supplement influenced your performance”, and their reason why. Answers have been translated from Portuguese by two of the investigators.

Participant ID	ΔTTC (s)	Q2	Reason
**Improved**			
9	-4.15	2	I felt more able to perform the 1-km in the determined time
24	-3.71	1	Today I felt tired, with pain in the legs from training. I tried to pedal sparing myself at the start.
7	-3.34	1	Because I know (or believed) that it contained an inert substance.
17	-3.09	1	Because of the fact I knew it was an inert substance.
19	-2.50	2	During the exercise, more or less halfway, I remembered the tablets that I had taken, I saw the colour and the shape of them in my head and imagined that they were making me more “powerful”
28	-2.49	1	I felt more muscular fatigue throughout.
26	-2.02	0	I didn’t feel a difference in relation to the first visit
3	-1.95	2	Because I believed the explanation of the previous experiments with athletes and patients.
6	-1.88	2	I put it in my head that the supplement would help me, I thought throughout the test that it was beneficial, however, I don’t know if it actually helped me.
30	-1.77	0	I didn’t see a difference from the first test with this time-trial, today I thought it was worse even.
10	-1.60	0	Because I always think I’ve been worse every time. Maybe because I thought I already knew the necessary effort and psychologically forced myself to the finish line.
**Did not change**			
25	-1.26	2	During the test I thought that if it is true that open-placebo works, then maybe there would be some improvement, but I also ended up thinking that maybe it wasn’t a placebo. Another thing that I thought was that maybe the team was expecting an improvement and I didn’t want to let them down, so maybe I gave my maximum to meet the expectations of the team.
11	-0.82	1	I didn’t feel much difference from the last test.
29	-0.39	1	I feel a level of fatigue similar to the first session.
13	-0.36	3	Psychologically I felt fitter.
1	-0.32	1	I went to sleep late and was tired.
20	-0.24	0	I never think that performance is influenced by supplements, I have no idea (parameter).
4	-0.15	2	Because I know that it has nothing chemical, I just believed in the study.
12	-0.07	0	I don’t think there was time for it to work.
15	0.06	1	I didn’t feel that the test was easier.
21	0.32	1	Because I believe that my performance is easily influenced/affected by other factors (nutrition, sleep quality, mood…).
18	0.53	1	Didn’t feel anything different.
22	1.12	0	If it was inert then it [performance] has nothing to be influenced by, I don’t think there was enough time for digestion also.
23	1.23	1	Initially I didn’t believe/know the placebo effect. I believe that after the pre-test explanation and the results presented, I may have changed my previous opinion which was that there is no effect. Curious about the results.
**Worsened**			
27	1.49	0	Because I was told that the drug was placebo!!
2	1.95	0	I felt much more tired than the first test.
14	2.07	1	I think the fact that the medic told me it was a placebo, psychologically I didn’t notice an effect.
5	2.79	0	I had the impression of being more tired than the first time I did the exercise.

There were two clear themes reported by the 4 individuals who worsened their performance (#27, #2, #14, #5), namely that they felt more tired (#2, #5) and had a negative expectancy regarding the supplement since it was a placebo (#27, #14). Of the 13 individuals who showed no change in performance, there were similar reports of an inability to notice any differences (#11, #29, #15, #18) and a lack of expectancy regarding the supplement (#20, #4, #12, #22) although there were a few reports of positive expectation (#13, #23) and mental imaging (#25).

## Discussion

This is the first study to show that exercise performance can be improved on average when athletes are openly administered a placebo supplement compared to a control session, providing evidence that the use of a placebo in a non-deceptive way can be ergogenic. On average, the open-placebo intervention resulted in a 0.7 s improvement in 1-km time-trial performance compared with the control session. Although this difference might appear small, this improvement likely represents a meaningful and practically significant difference in performance at the elite level. Nonetheless, individual variation in response seems to be high and some athletes may even experience decreased performance.

The current study showed that an open-placebo intervention can improve mean 1-km cycling time-trial performance. It is important to contextualise these results with regards to previous research using ergogenic supplements such as caffeine, which has been shown to benefit the same exercise task [31], although not beta-alanine [32] or nitrate [33]. The age and training status of our participants were similar to those employed in these studies, while further research has shown these ergogenic aids to be effective in females. The mean improvement in time-to-complete the time-trial with caffeine was approximately -2.3 s *versus* placebo and control, meaning the current results represent an improvement approximately 30% of that shown with this active supplement [31]. The non-significant changes following four weeks of beta-alanine or placebo supplementation were 0.1–0.2 s [32], while nitrate resulted in a likely harmful 0.4 s worsening in performance [33]. Individual analysis using a statistical approach [28] indicated that 11 individuals showed a worthwhile change in performance, equating to a 39% positive response. This compares favourably with acute ergogenic aids such as sodium bicarbonate, which we have shown to have a response rate of approximately 43% [34], and just slightly lower than the 54% positive response rate we have shown with caffeine [13]. Taken together, this study showed comparable group and individual effects of an open-placebo intervention on 1-km cycling time-trial performance to that of effective [35] ergogenic aids.

This is the first study to employ an open-label placebo intervention within an exercise setting although earlier studies have demonstrated its efficacy in a clinical setting for alleviation of symptoms of conditions such as irritable bowel syndrome [7], chronic low back pain, [8], allergic rhinitis [9, 10], depression [11] and cancer-related fatigue [12]. The 39% positive response rate to the open-placebo intervention in this study is lower than the 59% relief rate shown for irritable bowel syndrome [7] or 74% improvement in fatigue scores in cancer survivors [12]. In the current study, however, we employed a robust statistical method by which to determine responders and non-responders to the intervention [28] which employed stricter criteria for classification. Furthermore, we quantified an objective measure of exercise performance while previous clinical studies all determined subjective measures of disease symptoms. Subjective symptom scores may be biased by conditioning to give doctors (or researchers) positive feedback [36]. Taken together, the current data suggest that an open-placebo intervention, similar to those employed in clinical populations, is also effective in an exercise setting to improve performance.

Several individuals suggested they had positive expectations from the intervention, with two of those who improved reporting mental imaging to enhance their own belief in the supplement provided. For instance, participant #19 stated that “*During the exercise*, *more or less halfway*, *I remembered the tablets that I had taken*, *I saw the colour and the shape of them in my head and imagined that they were making me more ‘powerful’*”. Furthermore, participant #6 commented that “*I put it in my head that the supplement would help me*, *I thought throughout the test that it was beneficial*”. Interestingly, although some improvements were linked to the positive expectation instilled in the individuals, performance improvements were apparent even in certain individuals who did not report any optimism regarding the placebo capsule received. This was the case with participant #7, who claimed they did not feel the supplement had influenced their performance “*Because I know (or believed) that it contained an inert substance*”. Similarly, participant #17 also cited little influence, “*Because of the fact I knew it was an inert substance*”. Participants #24 and #28, who also improved performance, reported feeling more tired despite having replicated their training and dietary schedules, yet both improved performance by 3.4 and 2.4%. This demonstrates that performance improvements with placebo are not totally dependent upon expectation, corroborating previous research demonstrating improvements in pain tolerance [3] and cycling performance [19]. In fact, there is evidence that unconscious placebo effects (e.g., immune, hormonal and pain responses) may take place even if the individual’s expectations go in the opposite direction [3], which could be partially explained by classical Pavlovian conditioning. This study potentially extends this notion to open-placebo in the context of sports. It is also possible that some athletes improved performance partially through learning, which is a cognitively mediated placebo mechanism that could be as powerful as a conditioning procedure [37]. The systematic use of ergogenic supplements by our athletes may have elicited a placebo response for some of them in consonance with the idea that previous positive experience leads to reinforced expectations rather than to unconscious Pavlovian responses [38]. Twenty-six of the volunteers reported currently taking at least one supplement while supplement belief scores were high and that of users [25]. However, in the current study performance differences were not explained by supplement beliefs [25] or a positive personality [26]. Novel investigations should elucidate the exact mechanism(s) of open-placebo responses shown in the current study.

It is important to note that this intervention may not be effective for all individuals and, indeed, it is possible that it may even lead to negative or a nocebo response as demonstrated by four athletes in the current study who performed worse with open-placebo. Two athletes whose performance was impaired reported greater fatigue, participant #2 stating “*I felt much more tired than the first test*”, and #5 claiming “*I had the impression of being more tired than the first time I did the exercise*”. We did not determine whether these individuals felt more tired prior to the exercise which could have provided insight as to whether these feelings were already instilled, perhaps acting as a nocebo, or if they were a consequence of their poor performance. The other two participants who worsened performance cited a lack of expectancy, participant #27 citing no influence of the supplement “*Because I was told that the drug was placebo*!!” and #14 claiming, “*I think the fact that the medic told me it was a placebo*, *psychologically I didn’t notice an effect*”. Surprisingly, several individuals who reported similar negative feelings actually improved their performance, demonstrating a large inter-individual variation, which is likely influenced by numerous factors including the psychological response to the information provided, sociocultural background, and previous experiences with and beliefs in complementary interventions. We have previously shown a nocebo response when individuals believed they had ingested an inert substance [13], although not all studies corroborate these findings [19]. These data further highlight the intricacies and complexities inherent to the interplay between the investigator and the participant [38], and its resulting modulation of the psychosocial environment. Further research should investigate the intricate nature of expectancy and belief in response to open-placebo, and factors that contribute to any subsequent differences in performance outcomes despite similar prior expectations.

In the current study, we provided information to educate the individuals about the concept of placebo to ensure they understood what they were undertaking. Furthermore, we opted to instil a positive (though realistic) expectation around the open-placebo, suggesting that the placebo effect is a powerful tool and that although a positive attitude may help, it is not necessary and that the body may respond unconsciously, irrespective of belief [7]. This was done via a presentation given by a female medical doctor to increase a sense of confidence and professionalism in the information provided [23]. It is possible that we would have encountered different results without providing detailed information on placebo or inducing any positive expectations, although some research has shown that open-placebo works irrespectively of the provision of information and positive expectations [10]. Further research should investigate whether more neutral or reduced information, and delivery by a non-medical individual, also lead to performance improvements. Additionally, it would be of interest to determine the applicability of these data to different populations (e.g., males, elite athletes), time-trial lengths (e.g., 4-km or 10-km time-trial) and exercise modalities (e.g., running, resistance exercise). It is also important to determine whether responders and non-responders show consistent responses to open-placebo across repeated physical tests (*i*.*e*., within-individual variability), since it would allow exploring common characteristics that could predict performance outcomes and, hence, guide a personalized intervention. Finally, the underlying neurobiological mechanisms underpinning an improvement in performance should be investigated.

## Conclusion

An open-placebo intervention may be an effective tool by which to improve exercise performance since administration of an open-placebo protocol improved 1-km cycling time-trial performance in trained female cyclists. There was individual variation in the response and some athletes even experience decreased performance. Participant expectancy appears to play some role in the likelihood of gaining or losing a performance effect, but these factors do not entirely explain all changes in performance. Although open-placebo may constitute a novel, ethically acceptable ergogenic aid, some athletes may not respond or even perform worse (*i*.*e*., nocebo effect). Thus, open-placebo interventions should be carefully considered by coaches and practitioners, while further studies are warranted to determine underlying physiological and behavioural mechanisms for any changes in performance.

## Supporting information

S1 Data Data file. (XLSX)
